# Design and psychometric properties of the family support for older people questionnaire

**DOI:** 10.3389/fpubh.2023.1068839

**Published:** 2023-02-02

**Authors:** Soheila Shamsikhani, Fazlollah Ahmadi, Anoshirvan Kazemnejad, Mojtaba Vaismoradi

**Affiliations:** ^1^Shazand School of Nursing, Arak University of Medical Sciences, Arak, Iran; ^2^Nursing Department, Faculty of Medical Sciences, Tarbiat Modares University, Tehran, Iran; ^3^Biostatistical Department, Faculty of Medical Sciences, Tarbiat Modares University, Tehran, Iran; ^4^Faculty of Nursing and Health Sciences, Nord University, Bodø, Norway; ^5^Faculty of Science and Health, Charles Sturt University, Orange, NSW, Australia

**Keywords:** family support, home care, factor analysis, older people, psychometric properties

## Abstract

**Background and objectives:**

The population of older people is increasing across the world. Older people need care and support from their families to be able to live independently. This study aimed to design and evaluate the psychometric properties of the family support for older people (FSOP) questionnaire.

**Methods:**

In this instrument development study using a mixed-methods design, the psychometric properties of the FSOP questionnaire in terms of validity and reliability were evaluated.

**Results:**

The FSOP questionnaire consisted of 50 items in six domains. It was shown to have appropriate qualitative and quantitative validities (score > 1.5). The indicators of content validity (CVR > 0.62, ICVI ≥ 0.80, and SCVI > 0.94) and confirmatory factor analysis (indexes of χ^2^/df = 2.50, CFI = 0.96, GFI = 0.97, AGFI = 0.96, NNFI = 0.96, PNFI = 0.89, TLI = 0.96, and RMSEA = 0.06) were satisfactory. Cronbach's alpha coefficient for reliability was 0.94, indicating an optimal score.

**Conclusions:**

Healthcare providers and family caregivers are suggested to use the FSOP questionnaire for improving the quality of life of older people at home.

## 1. Introduction

It has been predicted that the number of older adults with low to high dependency levels will increase by almost a third by 2035 (1). Addressing the unmet care and support needs of the aging population and designing services and solutions centered around what older people need or want has become an urgent public health priority (2). However, the present gap in the workforce for the provision of care for patients suffering from long-term diseases requires initiatives emphasizing community-based care and partnership with family caregivers (3). When a society experiences demographic changes in terms of increases in the aging population, more people need to become volunteers and/or take on caregiving roles (4). Therefore, collaborative relationships and interaction between healthcare providers and family members can help smooth transitions of care to older people's own homes, leading to more patient-centered care (5).

Family caregivers are in the best position to support patients at home and coordinate healthcare services for them (6–8). The family has been recognized as the main support system for older people and plays an essential role in their health and wellbeing (9). Special attention to older people by family members is rooted in Asian cultural values, given that the family has always been considered the first line of support for older people (10, 11). The family is also considered responsible for fulfilling older adults' expectations and meeting their physical, mental, and emotional needs (12–15).

Family support can prevent hospitalization or the need to go into a nursing home (16). Older people usually prefer to live in their own home rather than in long-term healthcare settings (17, 18). It has been shown that older people who live in a familial environment and enjoy family support are healthier than those living in a nursing home or living alone (19, 20) and are more satisfied with their own life (21, 22). They often have better perceived health and self-esteem (23), leading to more successful aging (24–26).

The phenomenon of aging is experienced differently depending on country of residence, culture, economic condition, age group, and physical activity level. Accordingly, the phenomenon of support is defined and understood differently in different cultures and contexts (27, 28). In addition, family caregivers often face dilemmas in performing their caring responsibilities due to unclear boundaries and guidelines regarding support for older people in home care (29). The knowledge and skills of family caregivers for the provision of support in home care is improved through experience (30, 31). However, caregivers need education and support to reduce their own stress due to the knowledge gap regarding their roles, responsibilities, and accurately defining the dimensions of family support for older people in home care.

The development of a questionnaire with the consideration of norms and values that are appropriate to the cultural, religious, and social conditions of different societies is required for the development of strategies that can enhance family involvement in home care. It can also suggest a pattern for family caregiver roles in the provision of support in home care and be used for developing competencies to better fulfill their responsibilities. Given the lack of such an instrument in international literature, this study was conducted to design and evaluate the psychometric properties of the family support for older people (FSOP) questionnaire.

## 2. Materials and methods

### 2.1. Design

An instrument development study was conducted using a mixed-methods design consisting of qualitative and quantitative research approaches. The review of international literature and directional qualitative content analysis led to the definition of the research phenomenon (32). From this, perceived family support for older people in home care and the questionnaire's items were identified (13). Briefly, qualitative research was carried out to explore the perceived family support for older people from the perspectives of older people and their family members in the Iranian family context. The collected data was analyzed using the primary matrix developed based on existing international literature. Six main themes were used, upon which the items of the FSOP questionnaire were developed. The psychometric properties of the FSOP questionnaire in terms of validity and reliability have been reported in this article.

### 2.2. Psychometric properties of the FSOP questionnaire

The research was conducted from November 2020 until August 2021. The dimensions of the FSOP questionnaire and the related design matrix were as follows: instrumental (IN), financial (FI), affective (AF), therapeutic (TH), technological (TE), and social (SO). These dimensions defined the phenomenon of support for older people by family caregivers, helped with setting goals to measure perceived family support by older people, depicted the data analysis map, and developed a blueprint for producing items and scoring rules (33).

#### 2.2.1. Participants and sampling

The spread of the COVID-19 pandemic throughout the country led to the long-term closure of healthcare institutions, as well as rehabilitation and daycare centers for older people. Also, home quarantine was implemented, especially for the vulnerable groups of older people. Therefore, the imposed restrictions limited direct contact with older people for data collection.

For the recruitment of older participants, the family members of older people who participated in the qualitative research (13) from 30 cities across Iran were contacted *via* email using a convenience sampling method. Moreover, they were requested to nominate other families from their acquaintances using the snowball sampling method.

Recruitment was performed based on these inclusion criteria: older people aged ≥65 years; willingness to take part in this research; having no cognitive impairment; living at home; receiving support from family members.

An electronic version of the FSOP questionnaire was developed by the researchers. Distributing the electronic version of the questionnaire had the benefit of reaching older people with a greater geographical variation. Choosing the appropriate layout and font, displaying each question on a separate page, simplifying the questionnaire's items, and allocating enough time to provide answers were all considered in order to facilitate data collection from the participants.

The family members were asked to hand the FSOP questionnaire to older participants and request them to fill it out. The researcher could be contacted *via* email or telephone to answer probable questions and remove ambiguities during the data collection.

The sample size was estimated based on the number of items in the questionnaire. Between 2 and 10 participants for each item was recognized as sufficient (34). To avoid bias due to missing and/or incomplete data collection and to be assured of the adequacy of the sample size for confirmatory factor analysis (CFA), the highest accessible sample was recruited, which was 4.5 participants for each item. Therefore, the sample size was 235 older people.

#### 2.2.2. Face validity

To examine the qualitative face validity of the FSOP questionnaire, the principal investigator (SSH) conducted individual interviews with 10 older people to identify their perspectives on the understandability and simplicity of the questionnaire's items (33). These participants were approached by their family members *via* email and invited to take part in individual face-to-face interviews at times and places convenient to them. The interviews were mostly held in the older people's own homes or city parks. The participants' feedback and suggestions were included in the final version. For quantitative face validity, a five-point Likert scale was used to assess the questionnaire's items, from “strongly important” (score 5) to “not at all important” (score 1). The impact scores for each item were calculated and values over 1.5 were considered appropriate (33, 35).

#### 2.2.3. Content validity

Using the qualitative content validity method, ten experts in the fields of gerontology, family nursing, and questionnaire development were invited to review and give comments on the writing and presentation style of the FSOP questionnaire (36). The revised questionnaire was sent back to the expert panel for final approval. For quantitative content validity, the same expert panel was requested to assess the questionnaire upon which the content validity ratio (CVR), the item content validity index (ICVI), and Scale content validity index (SCVI) were calculated. According to the Lawche table, a CVR score above 0.62 indicates satisfactory content validity for the questionnaire (37). The ICVI determines relevance, simplicity, and clarity of items using a four-point Likert scale, and an ICVI score ≥ 0.80 is considered appropriate. A score greater than 0.90 for SCVI is considered appropriate (33, 35). The four-point Likert scale used to assess the ICVI was as follows: the sentence is complicated; the sentence needs rectification; the sentence is simple, but it needs rectification; the sentence is simple and clear.

#### 2.2.4. Item analysis

The preliminary evaluation of a questionnaire in its target population before its widespread use for data collection is item analysis. Item analysis is usually performed before evaluating the validity of a questionnaire's structure with a small sample of participants. Therefore, 30 older people were selected using the convenience sampling method and requested to complete the FSOP questionnaire (38). Each item in the questionnaire was evaluated for mean, standard deviation, correlation with other items, and the internal consistency of the whole questionnaire. The loop method was used for item analysis by which the reliability coefficient of the entire questionnaire was evaluated. If reliability increased with the omission of each item, the item had an effective role in coordination with other items, so it was entered into other stages of psychometric evaluation (33). The basis for deleting the item was whether the correlation of the item with the total item score was negative or <0.3.

#### 2.2.5. Construct validity

The structural validity of a questionnaire is assessed using factor analysis. It examines the relationship between items and identifies those items that are most related to each other and can form a single domain. In this research, CFA was performed, given that the aim of construct validity was to explore the relationships between the questionnaire's items based on predetermined factors or domains. Therefore, the questionnaire's items were assigned to factors based on theoretical expectations. The model fit statistics were used to assess the compatibility of collected data using the specified factor model (37), and confirm the goodness of the model fit (39).

Since the questionnaire used a Likert scale and the data distribution was far from normal, the robust version of the weighted least squares method was used, as the weighted least squares with robust standard errors and a mean- and variance-adjusted test statistic and other related methods were suitable for sequential scale data. The minimum acceptable factor loading was considered 0.3 (40). For evaluating the fit of the CFA, goodness of fit indicators were used along with the ideal score. Indices used in this study and their acceptable values to confirm the goodness fit of the model were as follows: x^2^/df ratio < 2, residual mean square error approximate (RMSEA) < 0.06; goodness of fit (GFI) > 0.90; and comparative goodness of fit index (GFI) > 0.90 (41, 42).

#### 2.2.6. Reliability

Internal consistency and stability were used for assessing reliability. Internal consistency was assessed using the calculation of Cronbach's alpha coefficient and composite reliability (CR). For stability, the intraclass correlation coefficient (ICC) was calculated *via* the test–retest reliability method. The closer the ICC was to one, the higher reliability level of the FSOP questionnaire. Test-retest reliability was evaluated through filling out the questionnaire twice within a 2-week interval by 30 older people (38). The values of Cronbach's alpha, ICC, and CR ≥0.70 were considered acceptable (41, 42).

#### 2.2.7. Responsiveness

The questionnaire should be sensitive to changes and be responsive. For this purpose, a hypothesis testing was performed to examine the total score of the FSOP questionnaire in relation to the participants' age using the independent t-test.

#### 2.2.8. Absolute stability

Absolute stability was assessed through the calculation of the minimum significant changes (MIC) of the questionnaire. For calculating MIC, the standard deviation of the base line score was multiplied by the mean effect size of 0.50 which was 17.18. The minimum detectable change (MDC) was also calculated using 1/96^*^SEM${\;}^{*}\sqrt{2}$. Also, the percentage of MDC was calculated using $\frac{MDC}{\bar{X}}*100$. The MIC should be larger than the MDC.

#### 2.2.9. Ceiling and floor effect

One of the criteria for interpretability is the desired ceiling and floor effects (35). The total score of the FSOP questionnaire was set between numbers from one to 100. Accordingly, the percentage of participants who scored either zero or 100 was determined. Also, the ceiling and floor effects were calculated separately for each domain. This index should be <20% (34), though there is no agreement among researchers, and some believe that it should be more than 15% (43).

#### 2.2.10. Feasibility

In order to evaluate the ease of the questionnaire, the average time to complete the questionnaire and the percentage of people who did not answer each item were calculated. The development of a lengthy questionnaire was avoided during item analysis and in the narrative stage.

### 2.3. Data analysis

Descriptive statistics were used for the description of the sociodemographic characteristics of older people. Inferential statistics were used to assess the psychometric properties of the FSOP questionnaire. All statistical tests were performed with a significance level of 0.05. The statistical software used for data analysis was SPSS 25 (IBM, Armonk, NY) and the Rosseel et al. package 2012 under R software (44, 45).

### 2.4. Ethical considerations

Ethics approval was granted by the ethics committee of Tarbiat Modares University (decree code: IR.MODARES.REC.1398.140). The participants and their family members were informed of the research aim being to assess the psychometric properties of the FSOP rather than to collect data about the current condition of family support for older people. The older people signed the informed consent form and sent it back to the main researcher *via* email. The designed questionnaire was distributed among participants who remained anonymous. Measures to ensure the confidentiality of the collected data were the restriction of access to the collected data, the anonymous sharing of data with research team members during data analysis, and saving the data on a password-protected computer.

## 3. Results

### 3.1. Demographic characteristics of the participants

In the present study, 149 women (63.7%) and 86 men (36.3%) participated. Of these, 143 participants (61.3%) lived with their families and 92 (38.7%) lived alone at home but received support from families. Table 1 presents the demographic characteristics of the participants.

**Table 1:** The demographic characteristics of the participants (*n* = 235).

**Variable**		***N***	**%**
Gender	Male	86	37
	Female	149	63
Education level	Illiterate	108	46
	Under diploma	57	24
	Diploma	35	15
	Academic	35	15
Marital status	Divorced	3	1.3
	Widow	88	37.4
	Married	144	61.3
Economic condition (self-report)	Excellent	3	1.3
	Good	76	32.3
	Moderate	132	56.2
	Poor	24	10.2
Medication use	No	37	15.8
	Yes	198	84.2
History of diseases	No	44	18.7
	Yes	191	81.3

“Unable to read or write” means Illiterate; “higher than diploma” means Academic.

### 3.2. Psychometric properties of the FSOP questionnaire

#### 3.2.1. Face validity

The item pool consisted of 62 items. During the qualitative face validity, six items were revised due to the perspective of the participants in terms of clarity. Also, the impact score of the items was reported as >1.5.

#### 3.2.2. Content validity

The CVR was reported as 0.94. Also, ICVI and SCVI were reported ≥ 0.80 and > 0.94, respectively. The CVI score of item 7 was 0.4. The item, stating “Without my children and other family members' help, it is not possible for me to clean the house,” had a CVR = 0.2 and overlapped with other items. Therefore, it was deleted. Items 10, 14, 15, and 52 had borderline CVR scores in comparison to the numerical value of the Lawche table. However, considering that they had acceptable CVI scores, they were kept in the questionnaire. Item 51 (“My children and other family members teach me how to use a bank card”), item 47 (“My children and other family members accompany me when using my bank card”), item 52 (“My children and other family members patiently repeat to me how to work with electrical devices and technological tools such as mobile phones, bank cards, computers, and the internet”), and item 53 (“My children and other family members explain to me the use of internet”) were all merged into item 58 (“My children and other family members help me patiently to use tools such as a mobile, computer, and the internet”).

The clarity and adequacy of the items were approved by the expert panel. Required corrections were made during qualitative and quantitative content validities. A 58-item questionnaire with a Likert scale of “always,” “often,” “sometimes,” “never,” and “rarely” was developed.

The questionnaire was first distributed to older people *via* a pilot study and the response process to the items was evaluated by the research team. This led to some modifications in the response scale. Some older people reported that they did not need support at some time periods. Therefore, the option of “I do not need support” was added to the Likert scale corresponding to the score of zero. The options of “rarely” and “sometimes” were found to be indistinguishable, so to remove this ambiguity the “rarely” option was deleted. Finally, the FSOP questionnaire as a 58-item questionnaire with the Likert scale of “always,” “often,” “sometimes,” “never,” and “I do not need” was developed.

#### 3.2.3. Item analysis

The research team evaluated the items based on the results of item analysis and made decisions regarding the need to maintain necessary items and improve the structure of the whole questionnaire. Accordingly, eight items were deleted. The coefficient after the deletion of items was 0.935 (Table 2). Therefore, the 50-item questionnaire was entered into CFA (Table 3).

**Table 2:** Item analysis of the questionnaire.

**Item**	**Mean score of the questionnaire if the item is deleted**	**Questionnaire's score variance in case of item deletion**	**Correlation of the item with the total score of the remaining items**	**Cronbach's alpha value in case of item deletion**	**Total number of items; Cronbach's alpha coefficient**
2	149.80	1,282.303	0.232	0.935	*n* = 58, 0.935
4	151.83	1,292.420	0.070	0.937	
13	150.27	1,285.857	0.123	0.936	
14	149.80	1,274.717	0.273	0.935	
21	149.47	1,288.326	0.297	0.935	
23	150.13	1,302.326	−0.002	0.936	
27	149.70	1,289.252	0.260	0.935	
48	151.67	1,335.471	−0.339	0.939	

**Table 3:** Family support for older people questionnaire.

**Items**	**Response**				
	**Score 1**	**Score 2**	**Score 3**	**Score 4**	**Score 0**
1. My children or other family members help me clean the house regularly	□Never	□Some times	□Often	□Always	□I don't need
2. My children or other family members help me with personal hygiene such as bathing	□Never	□Some times	□Often	□Always	□I don't need
3. My children or other family members help me with home appliances	□Never	□Some times	□Often	□Always	□I don't need
4. The health of home appliances is monitored by my children or other family members	□Never	□Some times	□Often	□Always	□I don't need
5. Unsafe home appliances are controlled and replaced by my children or other family members	□Never	□Some times	□Often	□Always	□I don't need
6. Inevitably, I am responsible for the maintenance of the house	□Never	□Some times	□Often	□Always	□I don't need
7. I get help from others to maintain my home appliances due to the lack of attention from my children or other family members	□Never	□Some times	□Often	□Always	□I don't need
8. My children or other family members provide facilities such as a toilet, installation of handles in the bathroom, installation of fences, non-slip surface at my home to prevent falling	□Never	□Some times	□Often	□Always	□I don't need
9. My children or other family members pay attention to the physical security of my home in terms of installing guards, secure doors, and alarm systems	□Never	□Some times	□Often	□Always	□I don't need
10. I depend on my children or other family members to prepare and cook food	□Never	□Some times	□Often	□Always	□I don't need
11. It is difficult to meet the current needs of my life without the help of my children or other family members	□Never	□Some times	□Often	□Always	□I don't need
12. My children or other family members regularly meet the current needs of my life	□Never	□Some times	□Often	□Always	□I don't need
13. My children or other family members support me financially in special situations and circumstances	□Never	□Some times	□Often	□Always	□I don't need
14. My children or other family members help me pay my current bills	□Never	□Some times	□Often	□Always	□I don't need
15. My children or other family members help me get my pension and salary	□Never	□Some times	□Often	□Always	□I don't need
16. My children or other family members help me with administrative and banking matters	□Neve	□Some times	□Often	□Always	□I don't need
17. My children or other family members respect me and do not blame me	□Never	□Some times	□Often	□Always	□I don't need
18. My children or other family members appreciate the hard work I put into them	□Never	□Some times	□Often	□Always	□I don't need
19. My children or other family members listen to my past memories and conversations	□Never	□Some times	□Often	□Always	□I don't need
20. I feel that my children and other family members care about me not being alone, especially at night	□Never	□Some times	□Often	□Always	□I don't need
21. My children or other family members understand my physical and mental condition	□Never	□Some times	□Often	□Always	□I don't need
22. My children or other family members try to cheer me up because my comfort is important to them	□Never	□Some times	□Often	□Always	□I don't need
23. My children or other family members are regularly greeted by telephone	□Never	□Some times	□Often	□Always	□I don't need
24. My children or other family members visit me regularly and ask for my help	□Never	□Some times	□Often	□Always	□I don't need
25. Damage to home appliances is remedied with compassion and urgency by children or other family members	□Never	□Some times	□Often	□Always	□I don't need
26. Children or other family members pay attention to my choices and interests (birthday, mother or father's day, holidays, and customs).	□Never	□Some times	□Often	□Always	□I don't need
27. My children or other family members pay attention to my rest and sleep	□Never	□Some times	□Often	□Always	□I don't need
28. My children or other family members teach me how to take care of my own health	□Never	□Some times	□Often	□Always	□I don't need
29. My children or other family members take me to the doctor for time-to-time check-ups and examinations	□Never	□Some times	□Often	□Always	□I don't need
30. My children or other family members pay attention and control my diet	□Never	□Sometimes	□Often	□Always	□I don't need
31. My children or other family members control my blood pressure	□Never	□Some times	□Often	□Always	□I don't need
32. My children or other family members provide first aid supplies to me	□Never	□Some times	□Often	□Always	□I don't need
33. My children or other family members teach me how to exercise at home	□Never	□Some times	□Often	□Always	□I don't need
34. My children or other family members prepare my medications and give them to me	□Never	□Some times	□Often	□Always	□I don't need
35. My children or other family members take care of me when I am hospitalized or am at home	□Never	□Some times	□Often	□Always	□I don't need
36. My children or other family members help me when I am sick or in need of immediate medical attention	□Never	□Some times	□Often	□Always	□I don't need
37. My children or other family members teach me how to take and store mediations	□Never	□Some times	□Often	□Always	□I don't need
38. When I have a disability or illness, my children or other family members take care of me	□Never	□Some times	□Often	□Always	□I don't need
39. My children or other family members help me to keep up to date with the latest news and communication tools	□Never	□Some times	□Often	□Always	□I don't need
40. My children or other family members accompany me when I use my bank card to allay my fear of making a mistake	□Never	□Some times	□Often	□Always	□I don't need
41. My children or other family members try to teach me how to use a landline phone and mobile phone	□Never	□Some times	□Often	□Always	□I don't need
42. My children or other family members teach me how to use electrical appliances, such as a washing machine, TV, etc.	□Never	□Some times	□Often	□Always	□I don't need
43. My children or other family members make it easy for me to work with electrical appliances by using labels and signs	□Never	□Some times	□Often	□Always	□I don't need
44. My children or other family members inform me about community issues and the prevention of dangers such as abuse and scams	□Never	□Some times	□Often	□Always	□I don't need
45. My children or other family members help me go on pilgrimages and to religious ceremonies	□Never	□Some times	□Often	□Always	□I don't need
46. My children and family members provide me with entertainment at home	□Never	□Some times	□Often	□Always	□I don't need
47. My children or other family members help me with my favorite pastimes	□Never	□Some times	□Often	□Always	□I don't need
48. My children or other family members help me with city and suburban travel	□Never	□Some times	□Often	□Always	□I don't need
49. My children or other family members pay attention to me at family meetings and sessions	□Never	□Some times	□Often	□Always	□I don't need
50. My children or other family members encourage me to connect with relatives and participate in the community	□Never	□Some times	□Often	□Always	□I don't need

#### 3.2.4. Construct validity

**Figure 1:**
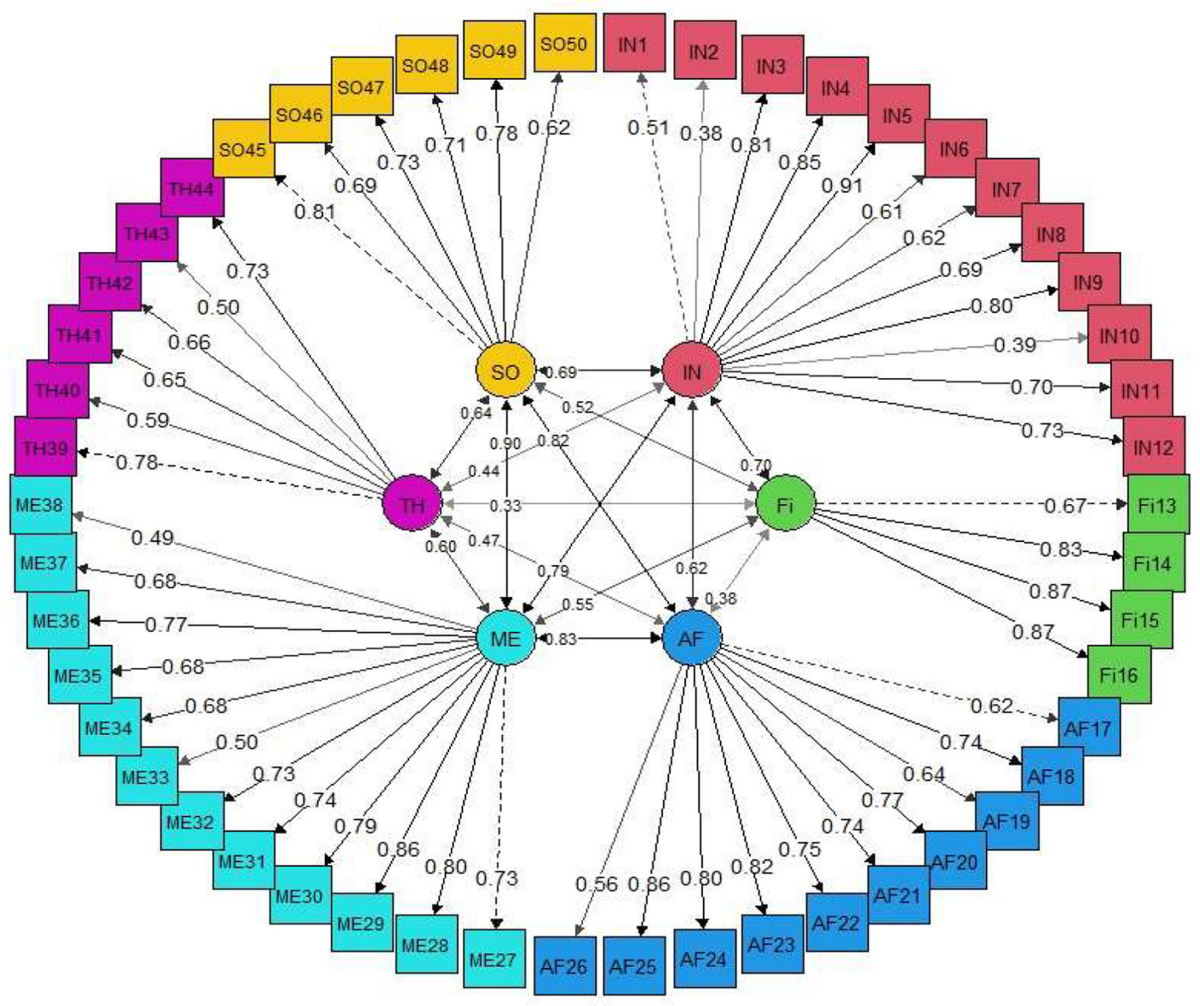
All indexes of $\frac{\chi^{2}}{df}=2.50$, CFI = 0.96, GFI = 0.97, AGFI = 0.96, NNFI = 0.96, PNFI = 0.89, TLI = 0.96 and RMSEA = 0.06 were measured and accepted in all domains. There were at least three observable variables for each latent variable and each observed variable measured only one latent variable of the agent.

**Figure 2:**
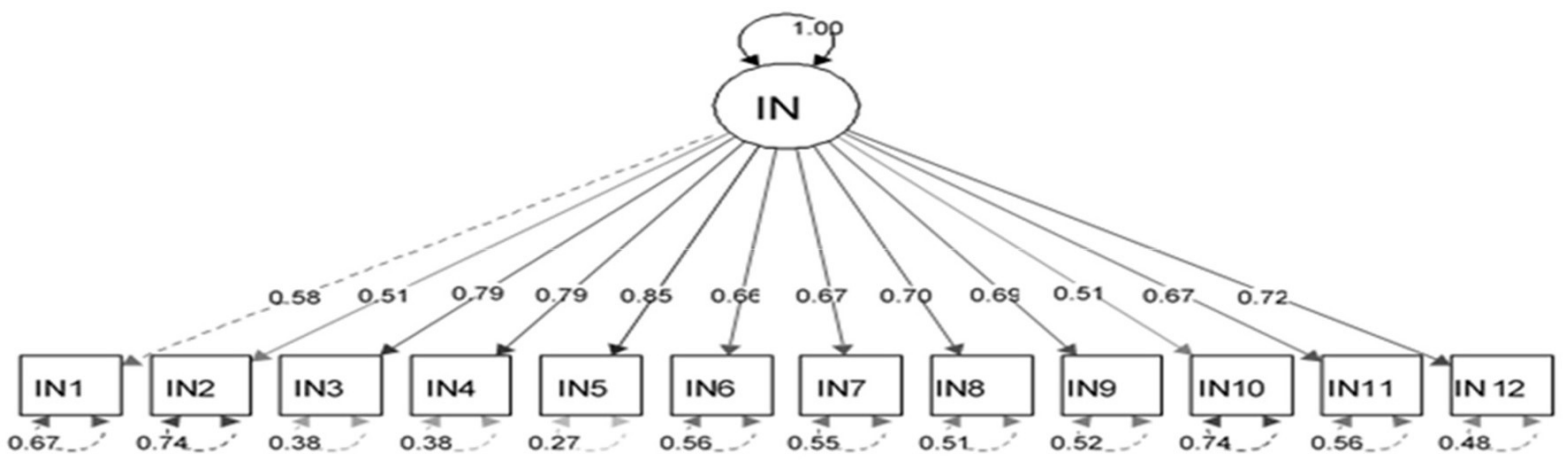
Confirmatory factor analysis model of the instrumental dimension of the family support for older people questionnaire. All items in the questionnaire showed a factor load >0.3, indicating appropriate metrics for the tool factor.

**Figure 3:**
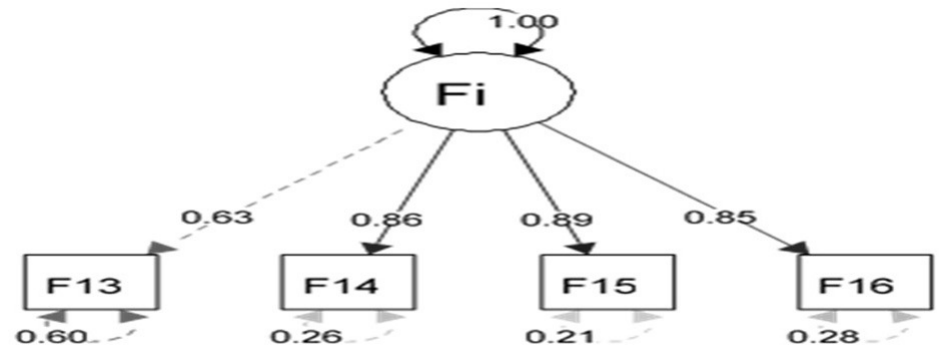
Confirmatory factor analysis model of the financial dimension of the family support for older people questionnaire. All items in the questionnaire showed a factor load >0.3, indicating appropriate metrics for the tool factor.

**Figure 4:**
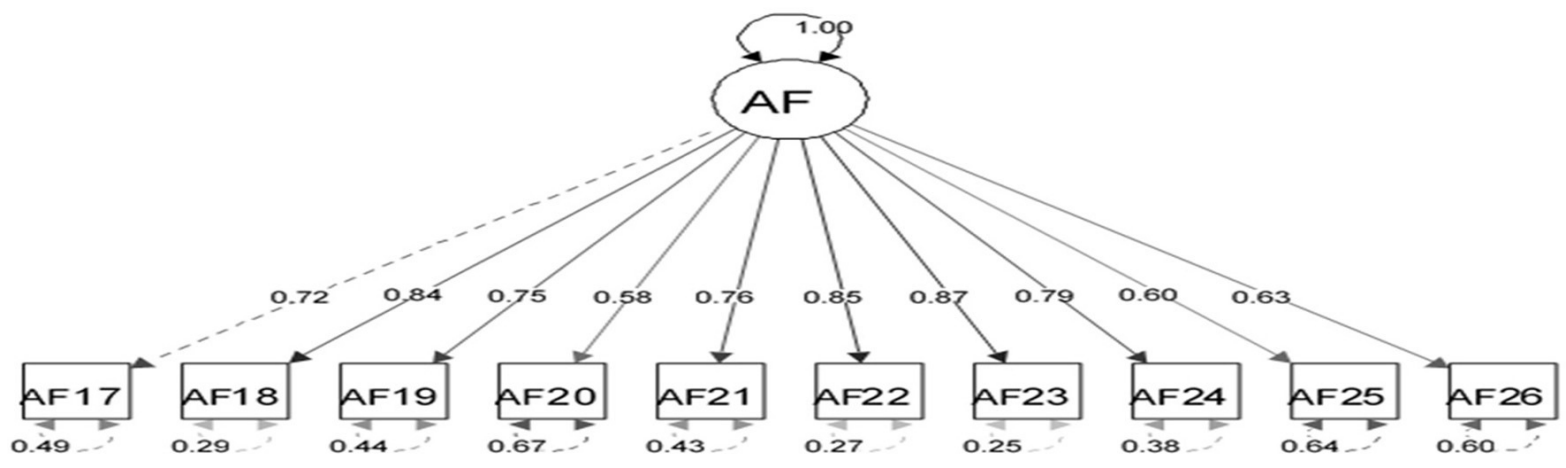
Confirmatory factor analysis model of the affective dimension of the family support for older people questionnaire. All items in the questionnaire showed a factor load >0.3, indicating appropriate metrics for the tool factor.

**Figure 5:**
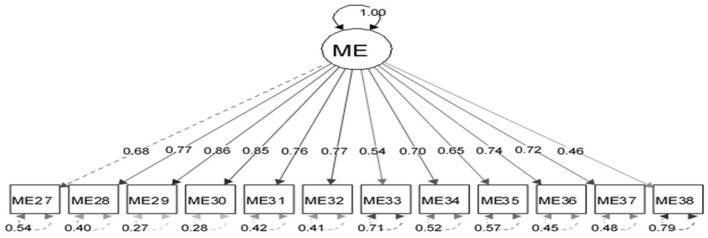
Confirmatory factor analysis model of the therapeutic dimension of the family support for older people questionnaire. All items in the questionnaire showed a factor load >0.3, indicating appropriate metrics for the tool factor.

**Figure 6:**
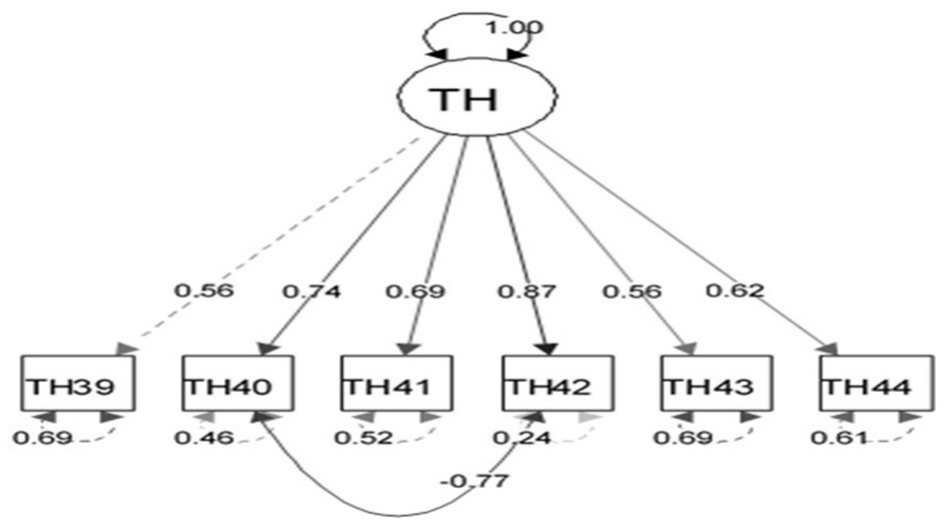
Confirmatory factor analysis model of the questionnaire on the technology dimension of the family support for older people questionnaire. All items in the questionnaire showed a factor load >0.3, indicating appropriate metrics for the tool factor.

**Figure 7:**
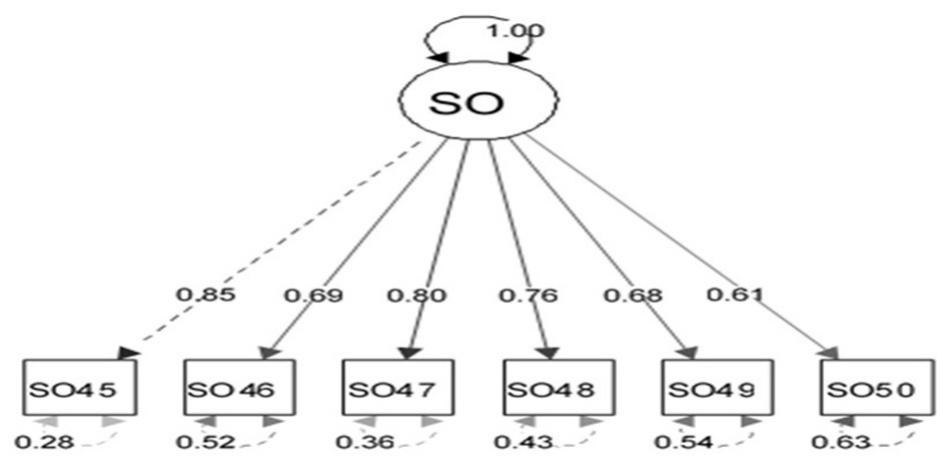
Confirmatory factor analysis model of the social dimension of the family support for older people questionnaire. All items in the questionnaire showed a factor load >0.3, indicating appropriate metrics for the tool factor.

The appropriateness of CFA was confirmed using goodness-of-fit indicators (Figure 1). These indicators, along with the ideal score, showed in general that all items had significant roles in the validity of the FSOP questionnaire. In particular, the important indicators of goodness of fit (CFI, TLI, RMSEA, $\frac{\chi^{2}}{df}$) for all models were within their acceptable ranges (Figures 2–7). The items were assigned to the following six domains: instrumental (IN), consisting of 12 items; financial (FI), 4 items; affective (AF), 10 items; therapeutic (TH), 12 items; technological (TE), 6 items; social (SO), 6 items.

#### 3.2.5. Reliability

Cronbach's alpha coefficient for the whole questionnaire was 0.94. The coefficients for the domains were as follows: IN = 0.87, FI = 0.82, AF = 0.86, TH = 0.87, TH = 0.76, and SO = 0.79. With regard to stability, ICC scores, with a 95% confidence interval for the whole questionnaire and for each domain, were reported in Table 4.

**Table 4:** The intraclass correlation coefficient (ICC) of the domains and the whole questionnaire.

**Factor/domain**	**Coefficient**	**Confidence interval**	***p***
Instrumental (IN)	0.96	0.90–0.98	< 0.001
Financial (FI)	0.96	0.92–0.98	< 0.001
Affective (AF)	0.89	0.74–0.95	< 0.001
Therapeutic (TH)	0.95	0.89–0.98	< 0.001
Technological (TE)	0.81	0.54–0.92	< 0.001
Social (SO)	0.89	0.75–0.95	< 0.001
Total	0.98	0.95–0.99	< 0.001

#### 3.2.6. Responsiveness

The results of the independent t-test (Table 5) showed a statistically significant difference between the participants' age and the mean score of the whole questionnaire (*p* = 0.032). Therefore, the results indicated the appropriate sensitivity of the FSOP questionnaire.

**Table 5:** Examination of the total score of the FSOP questionnaire in relation to the participants' age.

**Age group**	**Frequency**	**Mean score**	**Standard deviation**	***p***
≤ 75 years	175	135.87	33.65	0.032
>75 years	60	146.61	32.33	

#### 3.2.7. Absolute stability

The percentages of MDC in all domains except TH was close to or <30%, which were acceptable. Also, the MDC percentage of the whole questionnaire was 7.92 (Table 6).

**Table 6:** The absolute stability of the questionnaire in the six domains.

**Domain**	**Mean (standard deviation)**	**Intergroup correlation**	**Standard error of measurement**	**Minimum detectable change (MDC 90%)**	**MDC%**	**Result**
Instrumental (IN)	37.27 (11.37)	0.96	2.28	5.29	14.20	Acceptable
Financial (FI)	10.40 (5.46)	0.96	1.09	2.54	24.43	
Affective (AF)	33.04 (4.14)	0.89	1.38	3.20	9.68	
Therapeutic (TH)	34.90 (9.53)	0.95	2.13	4.96	14.20	
Technological (TE)	12.72 (6.21)	0.81	2.71	6.30	49.49	
Social (SO)	14.40 (5.43)	0.89	1.80	4.19	29.08	
Total	142.77 (34.37)	0.98	4.86	11.31	7.92	

#### 3.2.8. Ceiling and floor effect

The values for all domains reported were under 20%, indicating no ceiling and floor effects (Table 7). It showed the appropriate interpretability of the FSOP questionnaire, and the ability of the questionnaire to express true variability.

**Table 7:** The ceiling and floor effects in the domains.

**Domain or factor**	**Frequency**	**Floor effect (%)**	**Frequency of the highest value**	**Ceiling effect (%)**
Instrumental (IN)	1	4.0	3	3.1
Financial (FI)	10	3.4	43	3.18
Affective (AF)	0	0	30	8.12
Therapeutic (TH)	1	4.0	12	1,5
Technological (TE)	2	9.0	11	7.4
Social (SO)	0	0	24	2.10
Total	0	0	0	0

#### 3.2.9. Feasibility

The approximate duration of completing the questionnaire was between 15 and 20 mins. Also, the option “I don't need support” provided more choice to the participants and reduced the number of unanswered items.

#### 3.2.10. Scoring

The FSOP questionnaire had a five-point Likert scale. For the scoring of positive items, a score of 1 was given to the option “never,” 2 to “sometimes,” 3 to “often,” and 4 to “always.” For negative items, scoring was in reverse so that a score of 1 was given to the option “always,” 2 to “often,” 3 to “sometimes,” and 4 to “never.” Items 6, 7, and 11 had reverse scoring. In both positive and inverse cases, a score of 0 was given to the option “I do not need support.”

The score for each domain was determined through calculating the scores of related items of the domain. The total score of the FSOP questionnaire was determined through the summation of the scores for each domain according to a formula. The total score of the questionnaire varied from 50 to 200 and the level of family support was interpreted according to the qualitative quarters.

The following formula is based on the total raw score of each person divided by the minimum score that the same person achieved. For example, if for ten items the participant selected the option of “I do not need support,” he/she answered 40 questions with a minimum of 40 and a maximum of 160. If the total score of the tool was 100, this should be divided by 40 (the participant's minimum score), which achieves a score of 2.5, indicating a poor qualitative range of family support for the participant. If the participant selected “I do not need support” for eight items, their score ranged from 42 to 168. If he/she achieved a score of 110, divided by 42, the calculated score was 2.61, indicating the desired or good family support. According to qualitative quarters, a score of 1–1.75 denotes adverse family support, 1.76–2.5 is poor family support, 2.26–3.25 is favorable or good family support, and 3.26–4 is excellent family support. To facilitate the use of this questionnaire, the calculation of questionnaire scores can be easily performed using Excel.

## 4. Discussion

The research process led to the development and evaluation of the FSOP questionnaire consisting of 50 items divided into the following six domains: instrumental (IN), financial (FI), affective (AF), therapeutic (TH), technological (TE), and social (SO). First, 72 items were extracted from the data matrix. The scrutiny of the items by the research team led to the deletion of 13 items, and the 59 remaining items underwent evaluation for their psychometric properties, during which a further 3 items were added and 4 items were deleted. Therefore, 58 items underwent CFA, leading to the deletion of 8 items and 50 items remaining in the final questionnaire.

The process of evaluating the psychometric properties of this questionnaire complied with the Cosmin checklist as a consensus-based standard on the properties of instruments for the measurement of health statuses (35). The main characteristics and differences between the FSOP questionnaire and other similar instruments are discussed as follows.

In comparison to the FSOP questionnaire, the questionnaire by Wang et al. (46) on family support is focused on daily life, emotional support, and decision making using a Likert scale from never to always. Although friends and families were the main sources of support, instrumental support was exclusive to families in the Wang et al. questionnaire. In addition, the three important areas of family support only had three general questions.

The psychometric properties of the FSOP questionnaire were evaluated using valid scientific methods. Nevertheless, its adaptation to other cultures requires further investigations. In our study, frequent greetings were a type of emotional support. Xu et al. (47) assessed family support in terms of structural, instrumental, financial, and emotional support. Accordingly, structural family support was considered as whether participants lived with any of their children. Instrumental support was assessed by the question “Can your children accompany you to see a doctor?” the question for assessing child support was “Have you received financial support from your child in the last 12 months?,” and the emotional domain was weighed using the question “In general, do you think your children are respectful?” (47). No psychometric assessment was performed to ensure of the validity and reliability of these support domains. Xu et al. (47) also acknowledged that the evaluation of any kind of support with only one question was a limitation of their research.

Zimmer and Kwong's (48) instrument highlighted instrumental and financial support from children and other sources. Instrumental support was assessed through receiving assistance from a list of potential resources such as family members in four instrumental tasks, including food preparation, washing clothes, doing household chores, and shopping. Financial support was mentioned as any financial aid that added to older people's income. Respondents were asked if their children had sponsored them in the past year (48). In the comparison of this questionnaire with the FSOP questionnaire, commonalities in two domains and their content can be observed. However, the whole structure of the instrumental domain cannot be sufficiently evaluated through asking only one question. Also, construct validity of the FSOP questionnaire was investigated through CFA. The fit evaluation of the extracted model showed an acceptable fit with the dimensions of family support. In addition to evaluating the overall fit of the model, the model was fitted separately for each domain and an acceptable fit between the domains and the items was observed.

Komjakraphan et al. (28) developed a scale based on the results of exploratory factor analysis with three domains of: attention to daily life, financial and material assistance, and emotional and reassuring resources. These domains accounted for 57.03% of the total variance of family support for older people (28). Instrumental and financial constructs were two independent and important constructs that were merged after factor analysis, and the structure of accompaniment and companionship created the third domain (28).

Uddin and Bhuiyan (27) and Tselebis et al. (36) developed family support scales for older people, but they did not conduct factor analysis as the most important aspect of evaluating psychometric properties. The factor analysis process of the FSOP questionnaire showed that some items had more factor burden and were therefore more important in assessing the validity of the related domains. For instance, older people's concerns regarding continuing their independent life were highlighted. In general, older people prefer to stay at home as much as possible (22). The reduction of sensory, motor, and cognitive functions among older people often impairs their ability to perform activities of daily living and disturbs their peace of mind at home. Therefore, the safety and accessibility of household items should be assessed periodically (49). This could confirm the statement that a questionnaire should be developed based on the mental experiences of individuals that are rooted in their cultural constructions (50).

In the factor analysis of the technology domain of the FSOP questionnaire, the item regarding educating older people to use electrical appliances gained the most factor load. Older people face many obstacles using technology for performing daily life activities (51). They have to use various electronic appliances, indicating the impact of technology on their quality of life and their need for educational support from families (52). It has been shown that 98.8% of older people watch television in their spare time (53–55).

During factor analysis of the social structure of the FSOP questionnaire, the support of family members to assist going on pilgrimage trips and attending religious ceremonies was factor-loaded. Older people consider preparation for traveling as the duty of their children and prefer being accompanied by their children on such journeys (13, 50). In Muslim societies, a common spare time activity for older people is to go to the mosque to partake in religious rituals (55, 56). It can bring peace of mind and increase social interactions. Rahimi et al. (57) stated that older people were not supported by their families in leisure and recreational activities. The family support tool questionnaire developed in the Thai cultural context was shown to have an acceptable reliability and stability level. According to the Cronbach's alpha coefficients for reliability assessment, moderate to favorable correlations between subclasses were reported. Therefore, it was assumed that concepts measured by subscales were not completely distinct (28), which was converse to the FSOP questionnaire.

According to the Cosmin checklist, responsiveness and interpretability should be considered during the evaluation of a questionnaires' psychometric properties. Criteria for interpretability include the number of unanswered items, least significant change, distribution of total scores, and assessment of the ceiling and floor effects (35). Responsiveness, standard error, interpretability, ceiling and floor effects, and ease of the FSOP questionnaire were within acceptable ranges. Other similar questionnaires in international literature have not reported on these psychometric criteria (27, 28, 36).

Reliability assessment is an important part of the questionnaire's psychometrics evaluation. Most tool developers often assess internal correlation and stability of the questionnaire. Standard error calculation of the questionnaire has been neglected as an important component of reliability assessment in most questionnaires (58, 59).

### 4.1. Strengths and limitations

A strength of the FSOP questionnaire is the use of a mixed-methods design consisting of qualitative and quantitative methods for its development. Furthermore, there were no missing data, given that the principal researcher checked the participants' answers before entering data into the data analysis process. In addition, the psychometric properties of the FSOP questionnaire were evaluated based on various scientific criteria mentioned in the Cosmin checklist (35). The items of the FSOP are easy for older people to read and understand. In comparison to other questionnaires, the wide range of dimensions included in the FSOP questionnaire, such as instrumental, financial, affective, therapeutic, technological, and social, make it suitable for use in other cultures and contexts. However, further research should be conducted for the adaptation of this instrument to the culture of use. A limitation of this study was the probable inability of older people in understanding the instrument's content and items given the lack of presence of the main researcher during the data collection. However, the participants knew that they could reach the researcher *via* email or telephone and seek answers to their questions. Also, the feedback and suggestions by the older participants and the expert panel were included in the final version of the FSOP questionnaire to ensure the simplicity and understandability of its items.

## 5. Conclusion

The FSOP questionnaire covers various aspects of family support for older people in instrumental, affective, financial, therapeutic, social, and technology domains. It has successfully passed all stages of evaluation of psychometric properties and also goodness of fit for all six domains of family support. The FSOP questionnaire can be completed by both self-report and interview. This questionnaire has short and understandable items that suit older people's memory and cognitive conditions. The dimensions and items of the FSOP questionnaire can be used as a guide for developing educational programs to meet the educational needs of family caregivers with regard to support for older people in home care. It is suggested that healthcare providers and family caregivers use it for improving the quality of life of older people in their own homes.

## Data availability statement

The raw data supporting the conclusions of this article will be made available by the authors, without undue reservation.

## Ethics statement

The studies involving human participants were reviewed and approved by Tarbiat Modares University, Tehran, Iran. The patients/participants provided their written informed consent to participate in this study.

## Author contributions

SS, FA, AK, and MV: study design, conceptualization, data collection, data analysis, interpretation, and manuscript writing. FA, AK, and MV: study supervision. All authors contributed to the article and approved the submitted version.
